# How to Pick a Neuroprotective Drug in Stroke Without Losing Your Mind?

**DOI:** 10.3390/life15060883

**Published:** 2025-05-30

**Authors:** Joseph S. Tauskela, Nicolas Blondeau

**Affiliations:** 1 Human Health Therapeutics, National Research Council of Canada, 1200 Montreal Road, Ottawa, ON K1A 0R6, Canada; 2 CNRS, IPMC, Université Côte d’Azur, Sophia Antipolis, F-06560 Valbonne, France; blondeau@ipmc.cnrs.fr

**Keywords:** cerebral ischemia, neurotoxicity, neuroprotection, excitotoxicity, mult-electrode arrays

## Abstract

All human clinical trials evaluating neuroprotective therapeutics in cerebral ischemia have failed, casting a pall over the field which has not recovered. Numerous methodological issues in the performance of these trials were identified, with the result that current trials are now subject to higher degrees of rigor and transparency. Advances in re-canalization technologies now offer the hope that adjunctive neuroprotection can improve patient outcome. The evaluation of neuroprotection in preclinical animal models has also suffered from methodological issues, which has also been addressed, resulting in an improved performance of studies. This leaves the question of how to actually pick the most appropriate neuroprotective therapy for translation. Given the current limitations in resources, and the numerous strategies that have been proposed to take advantage of clinical and preclinical methodological improvements, we suggest that in vitro studies involving subjecting the most sensitive cells—neurons—to oxygen–glucose deprivation (OGD) can be used to resolve among the many possibilities. Specifically, a large body of evidence shows that successive increases in OGD durations (spanning the lethal/supra-lethal continuum) require increasingly ‘strong’ drugs and combinations to adequately protect neurons (criteria not met in clinical trials). Notably, as the OGD duration is lengthened, NMDA receptor (NMDAR) antagonists of increasing potency and dose are required to match this increasing severity. Under supra-lethal OGD conditions, cocktails composed of anti-excitotoxic antagonists with maximal potency and dose are required to achieve neuroprotection. We propose that this approach can serve as a strategy—a neuroprotective framework—to prioritize among the many possibilities that exist for neuroprotective therapeutics for translation. Specifically, utilize the OGD continuum to compare within-, between- and outside-classes of drugs, first alone and then in combinations, to identify the most efficacious drugs (‘head-to-head’ competitions to identify the ‘last man standing’). While the current state of knowledge strongly suggests that anti-excitotoxic approaches are required, this framework allows the integration of testing established and new therapeutics alike. This framework should include new technologies such as multi-electrode arrays (MEAs), which allow the evaluation of adverse effects of drugs alone, as well as if a drug truly provides functional neuroprotection, and not just survival. The neuroprotective framework provides a comprehensive strategy to eliminate ineffectual treatments, leaving only those modalities with the highest therapeutic index to be prioritized for translation.

## 1. Failures of Neuroprotective Drugs in Cerebral Ischemia

One in four people worldwide will experience a cerebrovascular accident/stroke, which comprise the second largest cause of death worldwide (annual mortality rate of ~5.5 million), and is expected to rise with increases in risk factors [1] and references therein]. All (>100) clinical trials aimed at protecting neurons by the administration of drugs during or shortly following cerebral ischemia have failed. As a result, judging by the decline in resources devoted to this field and other issues, the perception exists that neuroprotection in stroke may not be achievable.

In the first part of this review, we attempt to prove what the neuroprotection field has ostensibly already proved; that is, previous neuroprotective drugs were inadequate. It is ironic that so many investigators have lost faith in the concept of neuroprotection, because numerous retrospective analyses have identified so many issues in the performance of human clinical trials that this concept has not been disproved. Part of the disappointment in human clinical trials stems from the ostensible success of preclinical animal model data, giving rise to a translational gap. Yet again though, numerous issues have since been identified in preclinical investigations, resulting in overestimations of efficacy. Thus, because of the translational gap, as well as an emerging perception that the challenge and expense of identifying neuroprotective candidates may be too difficult to sufficiently advance in well-designed preclinical animal model studies, numerous suggestions have been made to improve neuroprotective prospects. Perhaps the major theme that has emerged is that the one target/one drug/neurocentric approach has been too restrictive. This is particularly the case with excitotoxicity, the preeminent target of preclinical and clinical investigations. However, just as for the neuroprotective concept, the excitotoxicity concept has also not been disproved in clinical or animal model studies (except under certain conditions to be defined).

In the second part of this review, we suggest a neuroprotective framework to identify neuroprotective therapies with the best chance of translation, by overcoming the deficiencies in previous approaches. Advances in reperfusion technologies will allow a more informed opinion to be obtained on the viability of the neuroprotection hypothesis. We propose that an in vitro neuron culture approach can be a very important part of the solution. The conventional approach almost always involves an in vitro approach to identify relevant biological targets, mechanism of action and proof of concept of a putative neuroprotective agent. What is perhaps less well recognized, though, is that a substantial in vitro literature exists regarding the prioritization of targets and therapeutics in neurons. We identify numerous parallels in neuroprotection between in vitro neuron culture, brain slices and in vivo animal models of cerebral ischemia, strongly suggesting that considering the translational pipeline at an earlier stage than usually considered—in vitro, utilized in a manner emphasizing prioritization—not only has considerable merit, but may actually form an integral component to the solution. Given the backdrop of extensive failure and abandonment in the neuroprotection in stroke field, a new approach is required, built on a historical platform, taking advantage of lessons learned and new technologies, so that perhaps investigators and investors will be more inclined than ever before to consider this ‘back to the future’ approach.

### 1.1. Human Clinical Trials in Neuroprotection: Methodological Issues and Solutions

Numerous methodological issues have been extensively documented in the performance of human clinical trials testing neuroprotection against stroke [2,3,4,5,6,7,8]. Issues identified include poor trial design, nonexistent/poor stratification of patients according to stroke severity and time of stroke onset, administration of drugs well beyond optimal time windows identified in preclinical studies, and poor or unknown pharmacokinetics and pharmacodynamics profiling resulting in inadequate dosing. From the perspective of timing of administration alone, almost all phase 2 and 3 clinical trials evaluating neuroprotection enrolled subjects ≥ 4 h after stroke onset (up to 2013) [9], despite strong preclinical evidence that appreciable neuroprotection is likely unachievable beyond this time-frame, particularly in the case of anti-excitotoxicity based treatments in both in vitro cultures and slices and in vivo [4,10,11,12,13]. It is crucial to understand that any one of these issues on its own can account for a trial failure, and therefore did not prove that drugs (or their targets) failed, with the possible exception of only the most recent trials. Even a bona fide neuroprotective therapy would have failed due to any one of multiple methodological failures. This distinction is important because clinical trial failures are often used as a primary basis for charting a new course in neuroprotection by pursuing alternative cellular signaling mechanisms and therapeutics (a familiar theme throughout this review).

Critical advances have been made in correcting human clinical trial methodological failings, with more recent trials adhering to these much more rigid guidelines. To this end, various iterations of STAIR (Stroke Treatment Academic Industry Roundtable) provided a framework to overcome failures identified in clinical trial design and in preclinical assessments, with the latest version now STAIR XI as of 2021 [14].

Many neuroprotective drug trials were tested before the recanalization era, dramatically minimizing potentially salvageable tissue and drug penetration to the infarct, so most drugs would have failed simply because of the lack of reperfusion (essentially a permanent ischemia) [14]. Mortality and disability is very high in most patients experiencing large-vessel occlusion (LVO) without revascularization [15]. This situation has now considerably changed. The use of IV recombinant tissue plasminogen activator (tPA)—intravenous thrombolysis (IVT)—has been implemented for over 2 decades, but is potentially hemorrhagic and can have low efficacy due to low rates of recanalization in large arteries. Although IVT is still recommended, endovascular therapy (EVT) now forms a mainstay treatment of LVO, with a higher recanalization rate, lower complication rate and longer temporal window of administration. However, importantly, functional outcomes remain suboptimal [15,16]. It has been estimated that approximately one-half of patients treated successfully with EVT nonetheless retain poor functional status (‘futile reperfusion’) [17,18,19]. Thus, providing neuroprotection before and/or after successful recanalization is still absolutely required.

### 1.2. Preclinical Animal Models in Neuroprotection: Methodological Issues and Solutions

The failures of clinical trials resulted in questioning why ‘everything works in animals but not in humans’ [20] (95% of published animal model preclinical studies on neuroprotection reported positive outcomes! [19]). These impressions that experimental evidence was overwhelmingly solid for single-mechanism drugs targeting neurons [21,22], yet failed in clinical trials, have also commonly been used as a further justification that the wrong drugs were pursued (and therefore that other multi-mechanism multicellular therapeutics should be pursued; discussed extensively below). However, the failures in human clinical trials were also the result of major methodological issues identified in the preclinical animal model testing responsible for drug translation. A cottage industry of retrospective studies has outlined critical concerns of preclinical data quality for at least two decades [7,23,24,25,26,27] in neuroprotection in stroke and, indeed, in other diseases [28]. Major failings include study design differences with clinical trials, with preclinical testing displaying different primary end points, unreported negative results, publication bias, inadequate blinding and randomization, insufficient attention being paid to quality criteria and statistically underpowered, retrospective studies and nonrepresentative animals (did not include both sexes, co-morbid and aged animals). Perhaps, not surprisingly, it has been estimated that only one-third to one-half of ‘positive’ experimental stroke studies actually demonstrated neuroprotection [29,30]. From the perspective of publication bias alone, it has been estimated that ~20% of experiments were unpublished, leading to overstatements of efficacy by 30% [31]. So, in essence, the preclinical foundation upon which neuroprotective drugs were brought to human clinical trials was fragile.

An extensively advocated solution to increase preclinical and translational research transparency and confidence has been preclinical multicenter randomized controlled animal trials [32,33,34,35,36,37,38,39]. The first multicenter preclinical stroke trial was performed in 2015 [40,41], with anti-CD49d chosen due to a diversity of publications regarding efficacy, and was followed by another study in 2016 (IL1-Ra) [42], which allowed greater heterogeneity between laboratories [41], and another study examining anti-interleukin-17A [38]. These studies showed modest effects on reducing infarct volume. In an extensive and comprehensive multi-center study (Stroke Preclinical Assessment Network [SPAN]) examining six different neuroprotective modalities combined with intravascular thrombectomy, only one drug (uric acid) met escalating testing models, even though ischemic events were short and treatments began immediately at reperfusion [43,44]. A major goal of these preclinical animal model trials was to establish that a multi-laboratory network can harmonize the evaluation of different cytoprotective modalities and, in so doing, encourage investment by the pharmaceutical industry and other drug developers. Nonetheless, the poor drug outcomes suggest a better selection strategy is required for translation to preclinical animal model trials. Notably, in the SPAN preclinical trial, of the six cytoprotective modalities evaluated, only two possessed neuroprotective properties.

### 1.3. Perceived Headwinds to Achieving Neuroprotection

Given the valuable insights obtained in improving human clinical and animal preclinical trials, combined with the success of EVT-IVT in achieving reperfusion in a majority of patients, the neuroprotection field would seem to be better positioned than ever for successful translation to treatment in humans. However, the perception that government and industry are nonetheless still reluctant to invest in clinical trials continues to persist, even with a large potential market [45,46], and despite the substantial advances made in addressing preclinical criteria testing of a neuroprotective drug [47,48]. Initially, the proportion of clinical trials sponsored by the pharmaceutical industry steadily rose, accompanied by declines in nonprofit/government sponsorship [49]. However, after so many failures, large Pharma cut their CNS program portfolios in half (from 2009 to 2014) [50]. Bringing a novel compound to market typically costs ~$1.4 billion (2013 dollars) [51]. The implementation of preclinical recommendations is also expensive; for instance, it was estimated in 2017 that a study of old (24-month) rodents of both genders could cost $100,000 [52]. The continued current deficiency of intellectual and financial investment at all levels of investigation—academic, government, clinical and, in particular, pharma—suggests that the substantial improvements and recent advances in understanding and overcoming logistical issues at both the preclinical and clinical levels have been insufficient to overcome the financial challenges necessary to change the momentum of the neuroprotection field.

Headwinds may exist also based on logistical concerns. It has been estimated that the penumbra occupies 30–40% of lesion volume within the first few hours of vessel occlusion [53]. If the penumbra truly represents the only rescuable tissue, then the absolute amount of tissue potentially salvaged by interventions may not meet the threshold to pursue as a corporate stratagem. The recognition that ‘time is brain’ may represent too high of a logistical hurdle for some investigators to believe that neuroprotection can be delivered in a timely enough manner perception (‘bolting the barn door after the horse has escaped’ [54]), particularly against the rapid and highly destructive excitotoxic component [55]. The absence of a neuroprotective therapeutic approved for humans has spurred appeals for a paradigm shift. It appears that a demonstrable inter-laboratory ability to evaluate candidates has been insufficient to galvanize re-investment in the neuroprotection field. Perhaps what is missing is proof that appreciable neuroprotection can be achieved.

### 1.4. Is the Best Neuroprotective Therapy for Translation Being Chosen?

Unfortunately, preclinical-based selection criteria for therapeutics to be translated appears to have been inadequate for quite some time. In a landmark study published two decades ago reviewing neuroprotection in clinical and preclinical cerebral ischemia, drugs given to stroke patients performed no better than drugs tested only experimentally, leaving the authors to conclude that “this raises the issue of whether we are in fact selecting the best drugs to carry forward to clinical trial” [56]. In fact, as of 2017, the FDA did not require demonstration of preclinical and translational efficacy in order to approve a drug [52], potentially lowering the motivation to perform comprehensive preclinical studies. It has been [53] noted that one-half of the 114 drugs reviewed in the O’Collins review [56] reported the negative human clinical trial data before preclinical animal data publication, if at all.

It has been recognized for some time that candidates may have been too weak to provide adequate neuroprotection [57]. Using the CAMARADES (Collaborative Approach to Meta-analysis and Review of Animal Data in Experimental Studies) database, an effect size of 31% (which was estimated to decrease to 24% due to publication bias) was concluded from 16 systematic reviews of interventions tested in animal studies of acute ischemic stroke involving >500 publications [31]. Similarly, a meta-analysis of preclinical studies performed in 2020 reported reductions in infarct size by an average of 24% (not corrected for the usual observed publication bias), with a therapeutic administered within 3 h of ischemia onset in greater than 80% of studies [29]. Translation of even this relatively low level of efficacy to humans would likely result in improved outcomes, although this may not be sufficient to overcome investment headwinds.

### 1.5. Proposed Themes Proposed for Picking a Neuroprotective Drug

In the absence of any solid guidance from clinical trials, considerable uncertainty exists, resulting in a variety of concepts as to how neuroprotection should be pursued. Various proposals can be categorized as follows:

(i) Re-testing of failed drugs has been advocated for, given that a substantial fraction of patients treated with EVT achieve successful reperfusion, especially for those drugs exhibiting acceptable safety profiles. Re-testing should include drugs that failed (‘back-testing’ [58]) in the era pre-dating IVT or EVT since, in the absence of reperfusion, a drug would in all likelihood not be efficacious; furthermore, this re-testing could take advantage of the increased rigor being adopted in preclinical studies [19,59,60].
(ii) Re-purposing of clinically approved drugs minimizes intellectual property concerns and improving drug accessibility in this manner to investigators should in principle allow broader participation in the field. Various organizations have made small libraries of drugs available to investigators, which are usually quite diverse, FDA-approved and off-patent [61,62], although this approach does not seem to enjoy widespread adoption.
(iii) The inability of the one-target one-drug approach to result in a clinical success has been a strong motivator for seeking alternative or additional solutions. However, single-target drug development is still encouraged, particularly against novel targets or against conventional targets in novel ways although, even then, single targets that act as a hub for multiple pathways seem preferred [14].
(iv) Endogenous neuroprotective mechanisms such as preconditioning and postconditioning have attracted considerable study [63], particularly if coupled with acute neuroprotective properties. Advances have been made such as in the field of neutraceuticals, in which the therapy acts as both a preconditioner and can exert acute effects against stroke [64,65].
(v) Pleiotropic drugs that target more than one neurotoxic signaling pathway have attracted considerable interest, using the rationale that cerebral ischemia activates numerous parallel and serial pathways [7,66,67], so a single drug with multimodal modes of action against the signaling activated by cerebral ischemia should be pursued [1,68]. In fact, the most recent STAIR XI in 2021 advocated for interventions exerting multiple mechanisms of action [14], again citing the continued failure of one-target/one-drug clinical trials [1]. As examples cited, hypothermia or antioxidants target various toxic cellular signaling pathways activated by cerebral ischemia. Some drugs have been designed to incorporate two active ingredients [69,70]. Off-target effects of multi-modal drugs may be subject to historical biases against ‘dirty’ drugs, partly due to concerns of increased toxicity. What is missing, though, in discussions about pleiotropism is whether the most critical signaling pathways activated by ischemia are being targeted, and in an effective enough manner. Instead, it is often implicitly assumed that targeting several components in the signaling cascade will result in synergistic or additive neuroprotection.
(vi) Combination therapy to target more than one neurotoxic signaling pathway can improve efficacy. A systematic review and meta-analysis from over 11,000 studies found that infarct size was reduced by 20% and 38% in focal cerebral ischemia by single and double therapy, respectively, which were adjusted to 14 and 28% when corrected for publication bias, in focal cerebral ischemia [71]. Limitations to implementing combination therapy include logistical issues (industrial, academic and regulatory). However, in principle, combination therapy allows different components of the neurotoxic signaling cascade to be targeted, at the optimal doses required to achieve efficacy; optimized combination therapy may allow synergistic neuroprotection, potentially decreasing the individual doses required, thereby reducing adverse effects. Strategies for picking the individual constituents vary considerably, raising the question of whether the selection process has been optimal.
(vii) It is important to evaluate the veracity of each of these concepts. But, the process of examination must not stop at the individual level. Given the overall relatively low levels of efficacy achieved in animal models of cerebral ischemia, and the considerable headwinds, it is necessary to go beyond demonstrating proof of concept to demonstrating superiority of concept. Out of all of these concepts—re-testing, re-purposing, single-target hubs, endogenous signaling, pleiotropism and combination therapy—is there one (or more) which offers the best chance of success? To aid in this decision, it is crucial to have a strategy to allow a more informed choice to be made about which concept offers the greatest potential for translational success.

## 2. A New ‘Neuroprotective Framework’ to Prioritize Therapeutic Selection

We propose a strategy—termed a neuroprotective framework—whose overarching goal is to identify the therapeutics offering the most translational promise at the earliest stage possible in the experimental process. Therefore, the goal is to identify therapeutics with the highest therapeutic index (ratio of efficacy/adverse effects). Two components are required to address the efficacy component of the neuroprotective framework. In this section, we first focus on pragmatic issues: which cell type in the neurovascular unit to prioritize, what biological dispersion to study and which test insult will be the most informative. Next, we then outline the strategy used to pursue identifying those concepts which will yield the most efficacious neuroprotection. This framework represents an important extension from typical practice in which investigators pursue a target and therapy in a silo-based manner. Instead, comparative studies examining the collective will allow identification of the most efficacious drug(s), thereby allowing back-identification of the most important biological target (‘diagnosis by pharmacology’). ‘Drugability’ and adverse effects from drugs of course require consideration in the translational pipeline, but should not detract from an initial emphasis on maximizing efficacy.

### 2.1. The Cells: Neurons

We suggest that a neurocentric approach remains the initial focus: once neuroprotection is obtained, then other cells types comprising the neurovascular unit (NVU) should be evaluated, although this does not represent mainstream opinion anymore. Based partly on clinical trial failures, many investigators have advocated for drugs targeting the entire NVU, and not just neurons, including white matter [1,21,67,72]. In fact, STAIR XI prefers the term cerebroprotection over neuroprotection to emphasize protection of the NVU over targeting just neurons [14]. Cell types other than neurons are affected by cerebral ischemia, and reperfusion following sufficiently severe ischemia can deleteriously affect the NVU [27].

However, it has been almost universally agreed for some time that neurons are the most sensitive and temporally reactive to the deleterious consequences of cerebral ischemia, which is why neuroprotection has formed the mainstay of therapeutic development for so long. Once a neuroprotective modality has been established, endothelial, pericytes and microglia should be targeted thereafter in research efforts [73,74], although it is recognized that cross-talk among different cell types may result in differential responses (and potentially adversely) to therapeutics [14]. In monocellular cultures, this trend also holds. It has also long been recognized that neurons are considerably more sensitive to ischemic-like insults than other components of the NVU, generally ranking in susceptibility as neurons > pericytes/endothelial cells > astrocytes [75,76,77]. (It has also long been understood that different sub-types of neurons exhibit varying susceptibility to ischemia in humans, animals, brain slices and monocultures [Refs. [78,79] and references therein]). Recognizing this differential vulnerability [74], the objective should be to define a neuroprotective strategy before focusing research efforts on targeting cytoprotection for the rest of the components making up the NVU. Subsequent steps include assuring that neuron-focused therapeutics do not interfere with function of other cells comprising NVUs.

### 2.2. The Biological Model: In Vitro

Given the current era of low-investment/low-confidence, an in vitro approach offers a higher-throughput, higher-content and lower-cost tactic to test among the myriad of concepts that have been proposed. In fact, the in vitro approach based on neuron cultures, arguably, has already offered the most detailed insight to date into strategic prioritization. It is recognized that in vitro preparations are usually used only to generate proof-of-principle validation of biological targets and therapeutics—and not to compare and prioritize among the many possibilities—before proceeding to animal model testing. However, as we now extensively document in the remainder of this review, strong congruity already exists in results obtained between in vitro and the more advanced in vivo models of cerebral ischemia.

### 2.3. The Test Insult: Oxygen–Glucose Deprivation (OGD)

Oxygen–glucose deprivation (OGD) performed on monocultures—composed either of neurons alone or, more commonly, with astrocytes and microgli also present—and brain slices has long been a mainstay as the prototypical in vitro model of cerebral ischemia. Some investigators believe that OGD primarily models the core infarct (but see below), and may therefore be less useful in identifying therapeutics targeting the penumbra [78]. On the other hand, OGD durations of at least 1 h are often required to initiate neurotoxicity, despite low oxygen levels, durations which are longer than typically used in slices and in vivo; however, plastic dishes typically used in in vitro experiments provide a reservoir for oxygen [80]. Another concern is that neuronal cultures do not undergo anoxic depolarization, which could be related to the presence of residual oxygen, and the lack of organized 3D architecture and anatomical complexity. However, in vitro and in vivo models of ischemia display high levels of congruity in their response to cytoprotective agents.

### 2.4. The Strategy: Utilize the OGD Continuum

The overarching strategy is to test neuroprotective modalities against increasingly harsh OGD or ischemic-like insults. This is usually accomplished by simply increasing the duration of insult, which we define as the ‘OGD continuum’. Each duration can have very different implications:

(i) Sub-lethal preconditioning: At the shortest end of the OGD continuum, a ‘mild’ OGD insult which is sufficient to stress neurons, yet not severe enough to exert any neurotoxicity, is a classical form of preconditioning neurons to withstand a subsequent otherwise lethal insult caused by OGD or other excitotoxicity-based insults [81]. However, how preconditioning actually protects neurons—typically defined as the end effector—and the robustness of this neuroprotection should be defined, particularly relative to the other concepts introduced above: essentially, this represents a comparison between endogenous neuroprotection offered by preconditioning versus the exogenous based concepts cited above (re-testing, re-purposing, single-target hubs, pleiotropism and combination therapy).
(ii) Lethal: Increasing the duration of OGD sufficient to cause ≤100% neurotoxicity represents a classical approach and typically comprises most studies. This level of neurotoxicity probably models a penumbral-like insult, representing tissue at risk most amenable to neuroprotection. Adjusting various other features can modulate the harshness of the insult. A more dense, almost confluent density of neurons or 3D neurospheres will succumb more easily to OGD than a lower density, due to higher cellular release of glutamate. Other variations include altering the ion composition of the extracellular media to more closely mimic the ischemic milieu [82,83,84], or altering the degree of oxygen deprivation. The majority of drugs that have been tested in neuron cell culture models of OGD fit within this category: the goal is usually to evaluate mechanism of action (MOA) and to demonstrate proof-of-concept of neuroprotection as a basis for translation to testing in animal models of cerebral ischemia.
(iii) Supra-lethal: Increasing the duration of OGD further results in an insult capable of killing neurons many times over. This type of insult has usually been performed much less frequently, since most drugs lose their neuroprotective ability under these circumstances. Instead of avoiding this type of insult, it should be embraced as a method to eliminate most neuroprotective modalities. Those therapies that are still neuroprotective against supra-lethal OGD (‘last man standing’) should be considered from an efficacy standpoint as the strongest candidates for translation to in vivo models of cerebral ischemia. As outlined extensively below, such approaches offer good correspondence with results obtained from other in vitro and in vivo systems employing increasingly harsh ischemia models. We propose that such an approach can form a bulwark in prioritizing a neuroprotective therapy amongst many other options.

## 3. The Neuronal Biological Target in the OGD Continuum

### 3.1. Lethal OGD: Excitotoxicity as the Neuronal Target

The most prevalent and extensively tested hypothesis of neurotoxicity caused by cerebral ischemia in vivo and OGD in vitro is a strong and early role for excitotoxicity in causing neuronal demise in cerebral ischemia. Ischemia in vivo or OGD in vitro results in cellular release of glutamate, which acts on post-synaptic glutamate receptors. Numerous studies show that NMDAR antagonists usually offer the highest degrees of efficacy, at least compared to other glutamate receptor, voltage-gated Ca^2+^ channel (VGCC) antagonists and antioxidants against lethal OGD (Ref. [85] and references therein). Furthermore, numerous downstream intracellular targets of glutamate receptors have demonstrated efficacy (reviewed in [86,87]). Pharmacologically mimicking OGD in vitro (an ‘OGD-mimetic’) by combining the Na/K ATPase inhibitor ouabain with TBOA to prevent cellular glutamate uptake also demonstrates neuroprotection using an NMDAR antagonist [88]). Other OGD-mimetic approaches include simply exposing neuron cultures to exogenous glutamate, NMDA or other glutamate receptor agonists, although this does not mimic the energy deprivation extremes of OGD/ischemia (see below).

Motivated by concerns expressed by clinical and preclinical researchers about the relevance of excitotoxicity, we tested a limited set of drugs exhibiting other mechanisms of action against lethal OGD. However, neuroprotection by diverse agents such as the Cl^−^ channel blockers DIDS/SITS [89], and an entire field of metalloporphyrin catalytic antioxidants [90,91,92,93], were surprisingly mediated by suppression of NMDAR-mediated Ca^2+^ influx. Similarly, other laboratories attributing neuroprotection by other drugs to non-excitotoxic means actually found an ability to block NMDARs, such as by the ryanodine receptor antagonist dantrolene [94] and the gap junction uncoupler carbenoxolone [95]. Before attributing efficacy to the primary effect of a drug, known mechanisms of action such as excitotoxicity must first be ruled out, thereby reducing confusion about what is actually a true neurotoxic target.

### 3.2. Sub-Lethal OGD: Preconditioning Is Anti-Excitotoxic

Besides investigating the usefulness of exogenous anti-excitotoxic agents, a different way of evaluating the veracity of the excitotoxicity hypothesis is to examine endogenous neuroprotection. In other words, how does preconditioning protect neurons from otherwise lethal OGD? Is this end effector different from investigator-driven exogenous sources of neuroprotection? Does protection extend to supra-lethal OGD? We reported that OGD-preconditioned neurons were completely protected against otherwise lethal OGD [96], and prevented the rise in extracellular glutamate (glutamate_ex_) [97] that normally accompanies OGD [98], in agreement with findings in other laboratories [99,100]. We extended these findings to show that preconditioning by exposure to the hyper-synaptic activators 4-aminopyridine and bicuculline to induce down-regulated synaptic scaling [101] also completely suppressed the rises in neurotoxicity [102] and glutamate_ex_ [103] during otherwise lethal OGD or exposure to OGD-mimetics.

Pursuing our neuroprotective framework strategy, we screened several dozen preconditioning agents against increasing longer durations of supra-lethal OGD. All preconditioners eventually failed to neuroprotect at different durations of OGD. Temporally, the point during OGD for which each preconditioner failed to provide neuroprotection correlated with a rise in glutamate_ex_. Notably, ischemic preconditioning delayed, but did not prevent, ischemic depolarization (which is associated with a massive glutamate_ex_ rise); prolonging the duration of the insult beyond the point at which glutamate_ex_ rose or ischemic depolarization occurred resulted in neurotoxicity, both in slices [104] and in vivo [105], indicating a ceiling of neuroprotection. Thus, preconditioners could be ranked according to their ceiling of efficacy; 4-aminopyridine/bicuculline preconditioning was identified as the ‘last man standing’ most able to withstand supra-lethal OGD [88]. This increase in glutamate_ex_ during OGD was the neurotoxic end effector, since the neurons could be ‘rescued’ with addition of an NMDAR antagonist just prior to the glutamate_ex_ rise for all preconditioning paradigms. Thus, a rise in glutamate_ex_ is the final toxic culprit in both naïve and preconditioned neurons, except in the latter case, this rise is delayed, suggesting essentially no effective difference in the mechanism of susceptibility to the post-synaptic excitotoxic component of OGD. Taken together, preconditioning ‘buys time’ by delaying cellular release of glutamate, the extent of which depends on the exact preconditioning paradigm, at which point an acute therapeutic must be administered [88]. Supra-lethal OGD has identified new concepts (ceiling, ranking and rescue) of most relevance to translation. Importantly, this has been a mechanistically driven strategy to develop a combination therapy of preconditioning/drug rescue, to identify the most neuroprotective regime possible and, at the same time, add clarity by ruling out many inferior preconditioners.

### 3.3. Supra-Lethal OGD: Continued Relevance of Excitotoxicity

**Figure 1:**
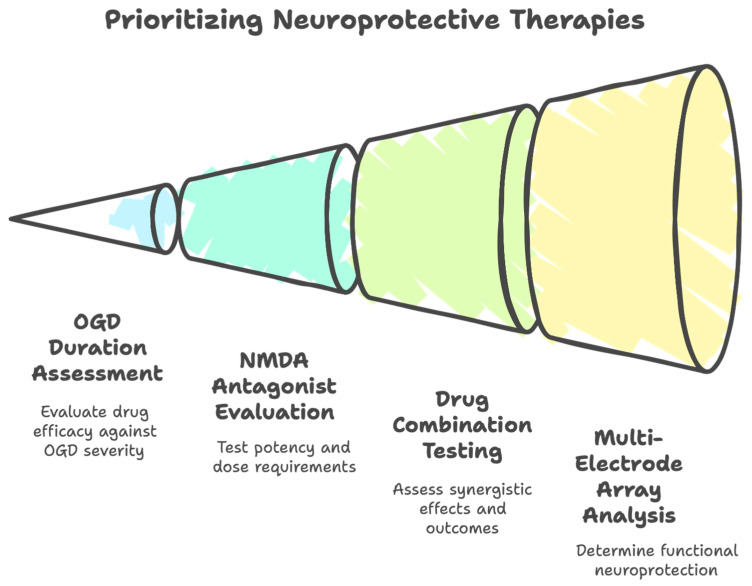
A schematic proposing a framework showing how optimal neuroprotection can be achieved, based on an extensive review of in vitro studies. When subjecting neurons to increasing durations of OGD—spanning lethal to supra-lethal consequences—NMDAR antagonists with successively higher potency and doses were required to provide neuroprotection. Longer OGD durations required the addition of other antagonists that blocked Ca^2+^ influx (AMPAR and VGCCs), each also with higher potency and doses. Neuroprotection was validated by Ca^2+^ assays and live/dead analyses, but now multi-electrode array (MEA) technology has progressed to the stage that will allow confirmation of preservation of neuronal network function by neuroprotective cocktails.

Supra-lethal OGD has been used to prioritize the strongest drugs in a limited number of laboratories performing in vitro studies now spanning over three decades of research. The general approach has been to first identify drugs able to protect neurons undergoing lethal OGD, and then, by instilling increasingly longer durations of OGD, identify drugs, doses and combinations able to still preserve neurons under these progressively hostile conditions. We now highlight in vitro findings from ours and other laboratories, with successively longer durations of OGD:

(i) Neurotoxicity caused by increasingly lethal OGD durations can be averted with more potent NMDAR antagonists. For instance, the more highly potent MK-801 is more neuroprotective compared to other competitive, uncompetitive and glycine-site NMDAR antagonists in neuron cultures and slice preparations exposed to OGD [106,107,108,109,110,111,112].
(ii) The concentration of the NMDAR antagonist must increase with progressive OGD duration [85,113,114,115,116]. In the case of MK-801, elevating its concentration—up to a remarkably consistent maximally effective concentration of ~10 uM—allows successively longer durations of supra-lethal OGD to be withstood [85,117,118,119,120]. The K_d_ for MK-801 may rise from 37 nM to < 500 nM in depolarized neurons [121], but why concentrations well above IC_50_′s of different classes of NMDAR antagonists are necessary to maximize neuroprotection is not fully understood. It has been suggested that NMDARs on cultured hippocampal neurons contain two populations with very different inhibition properties by MK-801, with a high (IC_50_ = 105 nM) and a low (IC_50_ = ~55 µM) affinity site [122]. Interestingly, adding another NMDAR antagonist with MK-801 improved neuroprotection [107,121,123], although this was not a universal finding [119].
(iii) Neuroprotection by even maximal amounts of MK-801 fails with further lengthening of the supra-lethal OGD duration [116,117,120,124,125,126]. This failure also extends to in vivo studies [127,128] and for other methods of blocking the NMDAR in vitro [121,129]. Neuroprotection with the maximal MK-801 dose also fails if the degree of oxygen deprivation is severe, even if glucose is present [130] or with longer term evaluation after a severe insult [113].
(iv) Antagonists of different receptors and ion channels must be combined to maintain neuroprotection at even harsher supra-lethal OGD. The most common combinations of antagonists are NMDAR and AMPAR with L-type voltage-gated Ca^2+^ channels, with the most common cocktail composed of MK-801 (10 µM), CNQX (10 µM) and nimodipine (2–10 µM) [111,114,116,117,119,121,124,131,132,133,134,135,136]. Blocking AMPAR’s may help prevent removal of the voltage-dependent block of NMDAR [111], enhancing neuroprotection.
(v) This cocktail fails to provide neuroprotection with longer durations of supra-lethal OGD [85,117,137,138,139]. Augmenting this cocktail with many other inhibitors (anti-apoptotic agents and other ion channel blockers) was similarly ineffective in different laboratories [85,117].
(vi) Neuroprotection can be achieved under extreme supra-lethal OGD conditions, but laboratories sharply diverge in the nature of the inhibitors used to supplement the anti-excitotoxic cocktails. Augmenting this MK-801/CNQX/nifedipine cocktail with TRPM7 or TRPM2 channel inhibitors protected neurons from supra-lethal OGD [117]. However, we [85] could not reproduce the finding that augmenting the cocktail with the TRPM7 inhibitor Gd^3+^ was neuroprotective [117]. The method of activation of TRPM7 in particular was attributed to the formation of ONOO^−^ from the reaction of O_2_^−^ and NO, which were blocked by the superoxide dismutase inhibitor MnTBAP and an nNOS inhibitor, respectively [117]. We also could not reproduce this finding with the nNOS inhibitor. As for MnTBAP, the mechanism of action is not as a superoxide dismutase [140]; instead, in a series of studies, we identified an off-target ability of MnTBAP (and other metalloporphyrins) to potently block Ca^2+^ influx through NMDAR’s, as well as from other sources, which was the basis for neuroprotection [90,91,92,93]. To determine if O_2_^−^ was truly involved, we augmented the MK-801/CNQX/nifedipine with various antioxidants, but this was ineffective too. Other studies report no role for TRPM7 in slices either [136].
(vii) Instead, we found that augmenting, manipulating and then changing the base MK-801/CNQX/nifedipine cocktail provided neuroprotection against successively longer durations of supra-lethal OGD. First, augmenting this cocktail with agents known to block Ca^2+^ influx (MnTBAP, ZnTBAP, verapamil and Ni^2+^) was neuroprotective. This suggested that the base cocktail MK-801/CNQX/nifedipine failed to sufficiently prevent Ca^2+^ influx (and likely accounts for why Aarts et al. [117] observed neuroprotection with the MnTBAP addition). Second, even longer supra-lethal OGD durations necessitated increasing the CNQX concentration and then exchanging CNQX with the more potent NBQX (less potent AMPAR antagonists were ineffective), at maximal concentrations. Third, more severe supra-lethal OGD necessitated exchanging MK-801 with a glycine-site NMDAR antagonist at maximal concentration, with the rank order of neuroprotection correlating with the rank order of potency of the glycine-site NMDAR antagonists. Figure 1 schematically summarizes the rationale used to identify the most neuroprotective cocktail ever identified in vitro [85].

### 3.4. Excitotoxicity Dominates the OGD Continuum

Taken together, both exogenous (i.e., antagonists/inhibitors) and endogenous (i.e., preconditioning) point to excitotoxicity as the neuronal biological target under lethal OGD in vitro. This view is even further strengthened when considering increasingly severe supra-lethal OGD insults, which require an increasingly stronger anti-excitotoxic based neuroprotective cocktail. It is clear that these types of drug cocktails will never be used therapeutically, but the goal was to understand the mechanism of neurotoxicity among many possibilities. Several implications can be drawn.

First, this does not mean that blocking specific sources of Ca^2+^ influx cannot be neuroprotective under certain conditions. However, context is important: cerebral ischemia is dynamically and temporally hostile, so a therapeutic that works under more benign conditions can fail to work under more harsh conditions.

Second, testing drugs which show efficacy under certain OGD-mimetic conditions—such as exogenous glutamate or NMDA—almost always considerably underestimates the dose required for OGD for reasons that are not completely understood (see discussion below), but which have profound implications for the neuroprotection field.

Third, the ‘strength’ of the insult must be matched by a commiserate increase in the ‘strength’ of the cocktail (potency, dose and drug combinations) so that neuroprotection can be achieved even under extremely severe conditions. The implications of a ‘strong’ requirement for such robust inhibition could be very important and suggest that anti-excitotoxic approaches in vivo and in humans should be considerably stronger than conventionally appreciated.

## 4. Role of Excitotoxicity in Other Model Systems

### 4.1. Excitotoxicity in Brain Slices and In Vivo

How do these results obtained with OGD in vitro compare with anti-excitotoxic approaches in brain slices and in in vivo animal models of cerebral ischemia? Cortical spreading depolarization (CSD) results in an increase in regional blood flow in order to meet increased energy demand, allowing enhanced clearance of extracellular metabolites. Repeated CSDs in the penumbra result in peri-infarct depolarization (PID). Under ischemic conditions, CSDs erupt spontaneously, causing hypo-perfusion, eventually resulting in expansion of the ischemic core, with lesion size correlating with the number and duration of SDs. CSD/PID exert extreme energy requirements on the brain, particularly the neurons, causing severe declines in local brain glucose levels; substantial energy is required to re-establish ionic gradients, reverse cellular swelling and intracellular organelle disruption. Recovery is possible with short but not longer durations of PID (reviewed in [141]).

The evidence for excitotoxic-based neuroprotection in brain slices remains divisive. It is well recognized that NMDAR antagonists can block SD evoked by numerous stimuli, such as high KCl or hypoxic SD, but become much less effective with CSDs during ischemia. The reason for this is not well understood but has been used as a basis for a consensus-based outright rejection of excitotoxic hypothesis [142]. One argument made against excitotoxicity is that glutamate_ex_ rises concomitantly (or soon after) with spreading depolarizations; however, this does not rule out an excitotoxic role for glutamate. NMDAR or AMPAR antagonists do not prevent anoxic depolarization (AD), but can reduce currents, the level of persistent depolarizations or the Ca^2+^ rise after AD or limit the AD amplitude. Cocktails containing both antagonists can prolong the latency to AD or prevent AD; neuroprotection fails with prolonged OGD, but increasing doses of NMDAR antagonists can re-establish efficacy (Refs. [85,142,143,144,145] and references therein). In acute brain slice and organotypic culture studies, another robust finding is that MK-801 (or other NMDAR antagonists) prevents neurotoxic rises in Ca^2+^ uptake during short (but lethal) durations of OGD or hypoxia (even in a modified ischemic media models [84,113]), which must be augmented by other Ca^2+^ channel blockers (particularly AMPAR antagonists) to preserve tissue exposed to longer durations [133,146,147,148,149,150,151]. These findings parallel the pharmacological profiles observed in lethal/supra-lethal OGD models in neuron cultures, suggesting that OGD in brain slices may be closer in severity to a supra-lethal OGD insult in neuron cultures. Similar high potencies/doses of cocktails effective in neuron cultures have never been tried in slices, to our knowledge. Overall, correlations exist between in vitro and in vivo studies, with NMDAR antagonists providing efficacy but failing under sufficiently severe conditions.

Numerous now-classical preclinical studies have shown that NMDAR antagonists reduce the Ca^2+^ increase, infarction or disability against various forms of cerebral ischemia in rodents or higher animals (reviewed in [87,152]); however, NMDAR antagonists failed in other animal model studies, suggesting that blocking NMDARs only may be insufficient (reviewed in [152,153,154]). MK-801 was generally more neuroprotective against mild but not severe global ischemia in rats (Ref. [155] and references therein), and also failed after severe transient forebrain ischemia [128]. Neither MK-801 or CNQX blocked the Ca^2+^ increase that accompanied the onset of anoxic depolarization within ~2 min of imposition of global ischemia in vivo [156], even using doses which blocked agonist induced Ca^2+^ increases. Overall, NMDAR antagonists can provide neuroprotection that nonetheless declines with lower potency/dose and increasing severity of insult.

### 4.2. Implications of Energy Deprivation on Anti-Excitotoxic Approaches

Taken together, a theme emerges. No matter the biological model under investigation—ranging from in vitro neuron cultures and slices to in vivo—an equimolar NMDAR antagonist can provide inferior neuroprotection against an ischemic insult (particularly with increasing severity) than against an exogenous NMDA or glutamate insult [85,106,113,156,157,158,159] (and numerous in vitro references cited above). A lack of understanding of this divergence may underlie skepticism about the role of anti-excitotoxic therapies in cerebral ischemia. This skepticism, in turn, has helped spawn the search for other causes of neurotoxicity in cerebral ischemia.

Even if anti-excitotoxic approaches are recognized, a key impediment to adoption may simply be a lack of understanding of why antagonists are so much more effective against exogenous NMDA or glutamate treatment than against the endogenous glutamate released in cerebral ischemia. Energy deprivation during ischemia is clearly an important component. As extensively reviewed, anti-excitotoxic approaches represent perhaps the strongest and most consistent evidence for neuroprotection against ischemia, but with diminishing usefulness as energy deprivation becomes more severe. An NMDA or glutamate insult does not typically cause nearly the energy deprivation that an ischemic insult does, particularly if the ischemia is prolonged. Bridging this gap somewhat, energy-compromised neurons are increasingly susceptible to exogenous glutamate [160,161,162]. A combined NMDA and AMPA receptor blockade was required for neuroprotection against exogenous glutamate in cultured neurons [163]. The requirement for increasingly aggressive drugs (potency, doses and combinations) helps preserve energy stores (Ref. [85] and references therein). A MK801/CNQX cocktail causes a decrease in cellular energy use during OGD, thereby inhibiting depletion of intracellular ATP [164], a critical factor in maintaining neuronal viability during energy stress [165]; even MK-801 has this effect in slices [166]. Thus, the increasingly aggressive cocktails used during supra-lethal OGD may be required to prevent any Ca^2+^/Na^+^ influx, thereby preserving ATP levels at survivable levels. A very recent study suggests that NMDAR/AMPAR blockade in organotypic cultures in turn suppresses glutamate release, by a newly identified plume-like release events [167].

To bridge this gap, more research is required in at least two major directions. First, it would be useful to continue studies examining anti-excitotoxic approaches using exogenous excitotoxins under energy-deprived conditions. Second, other non-excitotoxic mechanisms of neuronal demise may be activated under more severe ischemia. Indeed, the high concentrations required in individual drugs and cocktails under the most severe OGD conditions raises the possibility of non-specific effects. However, a counterpoint is neuroprotective efficacy correlated with increases in potency in each class of NMDAR and AMPAR antagonists constituting the cocktails. Furthermore, our extensive study ruled out numerous other mechanisms, and the highest degree of efficacy was still achieved with an anti-excitotoxic cocktail [85]. Nonetheless, not all biological targets were examined. Other targets that merit evaluation within this OGD-continuum based framework include (but are not restricted to) sodium–calcium exchangers (NCX), hemichannels (connexins, pannexins), acid-sensing ion channels (ASICs), volume-regulated anion channels (VRACs) and potentially other channels (reviewed in [168]) and ions such as Zn^2+^ [169], but see [170]. Others have suggested the presence of an additional Ca^2+^-permeable channel besides TRPM7, in the presence of Ca^2+^ entry antagonists, but this channel is blocked by Gd^3+^ [171], which was ineffective in our supra-lethal OGD model [85]. Answering these two fundamental questions may be important in determining the degree to which neuroprotection can be successful.

### 4.3. Proposed Refinements to Excitotoxicity

The poor tolerability profile of systemically administered NMDAR antagonists has motivated development of refinements to excitotoxicity, while maintaining the centrality of this concept [172,173]. The motivation has been to identify a neurotoxic component of NMDAR activation that needs to be selectively blocked, leaving normal glutamatergic function in the non-infarcted region of the brain intact. These theories are now outlined and are then placed in context with OGD-continuum findings:

(i) The source specificity hypothesis postulates that NMDARs are preferentially linked to downstream signaling mediators of excitotoxicity injury [174,175], since other sources of Ca^2+^ entry (VGCCs) were not toxic [176], although this has been proven otherwise [177].
(ii) A refinement of the source specificity hypothesis is to block neurotoxic signaling downstream of NMDAR activation, while still allowing normal basal synaptic function to be undisturbed [178]. Targeting death signaling cascades may extend the temporal window of activation, whereas NMDAR antagonists will have a much shorter temporal window. This has been the basis for development of PSD-95 peptides [179] and other peptides.
(iii) Extrasynaptic NMDARs promote death—and therefore should be preferentially blocked—while synaptic NMDAR activation promotes survival, and therefore should remain undisturbed [180,181]. Indeed, our preconditioning protocol of 4-AP with bicuculline to hyper-activate synaptic NMDARs ranked highest in providing efficacy against early supra-lethal OGD [101]. However, neuroprotection provided by peptides inhibiting synaptic PSD-95/nNOS signaling suggest that synaptic NMDARs can also be excitotoxic, a concept that has been confirmed [87]. This theory is predicated upon the assumption that activation of synaptic and extrasynaptic NMDARs cause equal loading of Ca^2+^ (emphasizing the location and not the Ca^2+^ loading), but this dogma has been strongly challenged [182]. Another key requirement is that targeting extrasynaptic NMDARs requires choosing weaker NMDAR antagonists at lower concentrations [183] (at odds with strong cocktail requirements). Memantine has attracted considerable attention due to preferentially blocking extrasynaptic over synaptic NMDAR currents [184].
(iv) The rationale behind Pathologically Activated Therapeutics (PATs) is to inhibit receptors that are excessively activated under pathological conditions, while having minimal effect on the target’s normal physiological activity. The uncompetitive open-channel NMDAR antagonist memantine has been proposed to be a PAT, since relatively lower concentrations of memantine block the effects of higher concentrations of NMDA due to more channels being open, while its fast off-rate allows normal transmission [183]. However, memantine is less potent than MK-801 in NMDAR-mediated current and neurotoxicity [185], and is therefore far less neuroprotective against supra-lethal OGD, even at maximal doses [85]. Since memantine will be repelled from the NMDAR during the membrane depolarization that accompanies ischemia, NitroMemantine was developed, which possesses an additional function of targeting an NO-generating group to the redox modulatory sites of NMDARs, outperforming memantine against cerebral ischemia [70]. A variation of PATs are ‘context-dependent’ NMDAR inhibitors which are more potent at acidic pH (such as exists in ischemic tissue) [186,187], one of which successfully completed a phase 1 clinical trial [188].
(v) Subunit-specific antagonists have been proposed: NR2B-containing NMDARs may be more toxic, possibly due to a more of an extrasynaptic location, than NR2A subunits located in synaptic pro-survival locations [189], but these subunits are located in both locations [190]. However, NR2B-containing subunits can mediate pro-survival signaling on their own [191] and, conversely, blocking NR2A-subunits can augment neuroprotection [182].
(vi) The Ca^2+^ overload hypothesis suggests that the amount of Ca^2+^ loading within neurons is the most important determinant of neurotoxicity, particularly with NMDAR-mediated entry [86,192,193], although see reference [173]. However, a concern with implementation of therapies preventing Ca^2+^ overload is that glutamate receptor activation (synaptic) is required for neuron survival, so blocking these receptors may harm naïve neurons (such as in non-infarcted regions), or prevent recruitment of endogenous recovery mechanisms in the injured region [54,152,194,195,196]. A dual issue has been suggested in which blocking only NMDARs may be insufficient so Ca^2+^ overload is not prevented in the core or near-penumbra, or can even be harmful by causing Ca^2+^ starvation and apoptosis further from the core and later time intervals (reviewed in [152]).
(vii) Additional or alternative target besides excitotoxicity have been suggested: between 1993 and 2001, 28 anti-excitotoxic approaches all failed in clinical trials so, perhaps inevitably (despite all of the methodological issues), given the intense focus on blocking excitotoxicity in clinical trials, many investigators have since come to doubt this hypothesis or, at the very least, suggested refinements [142].

### 4.4. Are Proposed Refinements to Excitotoxicity Relevant to Supra-Lethal OGD?

Out of all these possibilities, the Ca^2+^ overload hypothesis is by far the most concordant with investigations on the OGD continuum in in vitro preparations. Specifically, Ca^2+^ loading provides the strongest rationale of the necessity for combining antagonists to different receptors (mainly glutamatergic) and channels during supra-lethal OGD experiments. A recent series of studies employing a different approach came to the same conclusion. Ca^2+^ overload-induced mitochondrial dysfunction is a primary determinant of NMDAR-mediated excitotoxic injury [182]. This same group found that the higher susceptibility of hippocampal CA1 versus CA3 neurons in slices is due to a higher Ca^2+^ load and therefore mitochondrial dysfunction [197]. This group also showed that maximal L-type VGCC activation can induce high neurotoxic Ca^2+^ loading/mitochondrial dysfunction in a subset of neurons, which increases in proportion with aging, implicating Ca^2+^ loading in neurons as a general rule that is not restricted to only glutamate receptor activation [177]. Work using low-affinity Ca^2+^ indicators does indeed show that the degree of Ca^2+^ loading generally correlates with the degree of excitotoxic death [198,199]. A pleiotropic drug option that may prevent Ca^2+^ loading to the degree required is complestatin (3 µM), a peptide derived from *Streptomyces*, which is a noncompetitive antagonist against both NMDAR and AMPARs, that was more effective than MK-801 (10 µM) + CNQX (50 µM) in protecting cultured cortical neurons against supra-lethal OGD [200]. It would be useful to determine how effective complestatin is relative to the cocktail combinations we have used, but no follow-up to this drug has been published beyond 2002, as far as we are aware.

The dominance of data supporting the Ca^2+^ overload hypothesis does not necessarily entirely exclude the other possibilities listed above, but these are likely relevant only under less toxic conditions, since a common failing in application is insufficient potency and/or dose employed. It is also noted that a common theme in the above options is that only one aspect of NMDARs should be blocked, and each or all may be appropriate under lethal OGD (<100% neurotoxicity). However, in stroke, the build-up of glutamate would overwhelm all glutamate receptors [87]. The synaptic/extrasynaptic debate appears less relevant to supra-lethal OGD in neuron cultures or OGD in slices because conditions favoring this type of blockade—lower potency/dose of certain NMDAR antagonists such as memantine—to shift the ratio to higher toxic:nontoxic NMDAR-mediated Ca^2+^ influx will be insufficient. The subunit-specific hypothesis largely fails for the same reason, which is a lack of sufficient efficacy. Concerns that blocking glutamate receptors will not allow recovery in an injured region are likely irrelevant during the acute phase of ischemia, when it is clear that Ca^2+^ influx must be severely curtailed; at later time intervals, antagonists will likely no longer be present. Finally, the failure of anti-excitotoxic therapies have been due to methodological issues in trials, as well as an inability to achieve desired potency and doses (given the poor adverse effect profile for systemically administered drugs). This does not discount the possibility of considering non-excitotoxic approaches, but these must be considered in parallel and relative to existing evidence.

## 5. The Path Forward

### 5.1. Achieving Neuroprotection with Re-Canalization

Different approaches for systemically applied adjunctive neuroprotection (or cerebroprotection) are envisioned with EVT ± IVT. One approach is to systemically apply a therapeutic in concert with re-canalization in a hospital setting [7,15,201]. As of 2022, 15 randomized clinical studies examined neuroprotectants in which all patients had to receive EVT ± IVT, along with 15 more in which a subset of patients received EVT ± IVT [202]. A recent example is the PSD-95 peptide inhibitor nerinetide (TAT-NR2B9c) [179], which failed in the recent ESCAPE-NA1 [203] and ESCAPE-NEXT clinical trials. Interestingly, the failure of nerinetide has also not been interpreted as one of insufficient potency or efficacy; instead, the authors noted that the effect size improved with earlier treatment of patients (unpublished, but referred to in [204]). However, TAT-NR2B9c provides relatively weak neuroprotection in vitro (which we have confirmed; unpublished data), which can be overwhelmed with a more severe insult [205].

A second approach is to apply a therapeutic prior to hospital admission (field administration, or mobile stroke units) to ‘freeze the penumbra’ to extend the time window in patients likely to be candidates for thrombectomy [14,206,207,208]. A challenge is the lack of stratification in a pre-hospital setting to identify those patients most likely to benefit from treatment, although subsequent imaging in hospital may address this issue retrospectively. An important consideration is local pharmacokinetics, since drugs must rely on passive diffusion from healthy areas to reach the ischemic area: these distances depend on a number of factors but are quite limited. This will be an issue particularly in larger brains such as in humans, since much less infarcted area may be reachable (compared to small animals used in preclinical testing, adding to the list of potential translational roadblocks) [209]. The feasibility of achieving rapid administration was successfully demonstrated by the FAST-MAG clinical trial, but which failed [210]. This failure is perhaps not surprising, despite a pleiotropic mechanism being identified [211]. Mg^2+^ exerts a voltage-dependent block of NMDA receptors [212] and can be neuroprotective against an NMDA insult in vivo [213] and in vitro [214,215], although the level of neuroprotection is quite poor in vitro even under lethal OGD. Plasma membrane depolarization, such as through concomitant activation of AMPARs or VGCCs, will relieve the voltage-dependent block of NMDA receptors that accompanies cerebral ischemia [87]. In fact, in a review of nine studies, magnesium failed to provide neuroprotection in ~40% of preclinical animal model experiments [216], and the FAST-Mag protocol did not improve outcome after permanent MCAO [217] or global ischemia [218] in rats. Interestingly, among the reasons speculated for the failure of the FAST-MAG trial (including perhaps inadequate target engagement), low potency by Mg^2+^ was not included; instead, the failure of this single drug was interpreted as requiring pleiotropic or drug combinations [219]. A phase III clinical trial with nerinetide is ongoing with ambulance administration (FRONTIER: Field Randomization of NA-1 in Early Responders), with initial results suggesting improved outcomes, which was more pronounced in patients with confirmed ischemic stroke and subsequent vessel recanalization [204], hinting that a more rapid delivery may overcome lower potency. While encouraging, any clinical success should be regarded as a beginning, and not a finish, since continued pursuit of higher therapeutic indices will decrease disability and mortality. Importantly, a different poly-arginine peptide (within the same general class of cell-penetrating peptides) as nerinetide has shown higher efficacy against excitotoxicity insults in vitro and cerebral ischemia in in vivo animal models [220,221], so results of a phase II clinical trial should be instructive as to the importance of potency and target.

### 5.2. A New Chapter: Timing of Delivery an Essential a Trade-Off

The degree of efficacy that can be achieved with systemic delivery of a drug is essentially a trade-off between rapid delivery (pre-hospital) versus a more delayed delivery that is combined with EVT ± IVT (hospital). In the former case, delivery is the most rapid possible, but efficacy can be limited since the infarct is not perfused and instead relies on diffusion and collateral perfusion. In the latter case, drug delivery to the infarct is enhanced with recanalization. In either case, though, systemic delivery necessitates larger doses, which is limited by the dose/potency of the drug that can be achieved to avoid side effects and is subject to peripheral drug metabolism or excretion by liver and kidney. Importantly, adverse effects resulting from systemic administration of drugs is a major problem preventing achievement of maximal efficacy. In the context of the current discussion, it cannot be overstated that preclinical doses required for neuroprotection by antagonists of NMDARs could not be achieved in patients due to adverse effects, sometimes by orders of magnitude [4,55,222,223]. Crucially, a study determining the therapeutic ratios of NMDAR antagonists (and others) against permanent focal ischemia in mice found a correlation with those that failed in clinical trials [223]. This has profound repercussions, for the inability to achieve the dose (and potency) required is sufficient on its own to fail a drug.

An appealing third option emerging from preclinical animal model studies is to perform catheter-based intra-arterial delivery of a drug. Intra-arterial delivery of neuroprotective agents via the guide catheter already in place for mechanical thrombectomy should allow targeted delivery of the required (higher) dose/potency directly to the infarct, maximizing the potential efficacy, while limiting systemic exposure [224]. The gains in efficacy expected from this approach will be offset somewhat by the delay required for delivery in hospital. However, the fact that recanalization can be achieved with EVT in some patients even up to 12–24 h after stroke onset suggests salvageable tissue exists that would be amenable to neuroprotection, particularly if closer to stroke onset. In vivo animal models have been developed to allow intra-arterial delivery of magnesium sulfate, verapamil (Ca^2+^ channel blocker), ubeluzole (NMDAR antagonist) and targeted hypothermia with cold saline [225,226,227,228,229]. Do these represent the best candidates to test? Taken together, this approach likely represents the best option to achieve the high potency, dose, and combinations required, and should be vigorously pursued, but on drugs that have been subjected to a prioritized framework such as the in vitro approach that we have advocated for in this review.

In this scenario, establishment of reperfusion will eventually wash a drug away from the infarct area, unless intra-arterial delivery is maintained for some period of time, so it may be advantageous to consider drugs which have some neuronal adherence/internalization qualities, such as with antagonists having slower off-rates, or by TAT-peptides or cell-penetrating neuroprotective peptides [221] or other drugs that enter neurons. Perhaps the best-case scenario is a two-application approach: that is, provide a neuroprotective drug treatment in a pre-hospital setting—to ‘freeze the penumbra’—followed by administration of a drug in a hospital setting to take advantage of intra-arterial delivery. Different drugs or therapies could be provided within each setting—with therapeutic indices established in a dose response manner in in vitro testing—to maximize their respective advantages.

### 5.3. Determining the Therapeutic Index In Vitro

We have proposed a neuroprotective framework as an in vitro strategy to prioritize the most efficacious neuroprotective modalities for translation to animal model studies of ischemia. We suggest an in vitro approach analogous to that previously employed in vivo, in which an MCAO ischemia model was used to evaluate efficacy, and a behavioral test to evaluate side-effect liability, to produce the therapeutic indices of drugs [223]. Advantages of an in vitro approach include minimizing animal usage and costs, building on an extensive experimental groundwork, ability to incorporate testing of a variety of drugs, higher throughput and capacity and taking advantage of new technologies to provide high information content.

It is recognized that important components of the in vivo brain are not replicated by in vitro neuron cultures, or even brain slices, due to differences in architecture, cell types, cell distributions, maturity and numerous other factors. Indeed, these differences no doubt have contributed to in vivo-based investigators often ignoring in vitro models as a potential aid in solving neuroprotection in stroke. If these differences are sufficiently severe that in vitro OGD models are completely irrelevant in identifying targets and drugs, recall that the neuroprotective framework is composed of both the system/model (in vitro neurons subjected to supra-lethal OGD), but is also a philosophy of stress-testing neuroprotective modalities to identify only those with highest therapeutic index. However, as exhaustively outlined in this review, remarkable congruity exists in results spanning from in vitro to in vivo systems, suggesting a high degree of relevance in not only pursuing, but also in identifying, highest priority drugs. Excitotoxicity remains a remarkably consistent finding as a high priority target, and the principles comprising the neuroprotective framework not only allow but mandate putting each hypothesis to rigorous testing and comparisons.

For the efficacy component of the therapeutic index, in this review, we outline a philosophy and a framework to utilize in neuronal preparations. The overarching concept or philosophy has been to determine when and why therapeutics fail by stress testing them; only by understanding why therapeutics fail can potential remedies be developed rationally and systematically. This ‘success from failure’ philosophy may represent a different philosophy than usual, since usual practices to date have resulted in a dismal ‘failure from success’ outcome. We introduced the concept of the OGD continuum, in which the severity of OGD was successively increased, necessitating establishment of new parameters necessary to establish neuroprotection. For lethal OGD, numerous modalities provide neuroprotection, and this has doubtless formed the backbone of many drugs proceeding to animal model experiments. However, under supra-lethal OGD, ‘head-to-head’ comparisons revealed that almost all of these approaches failed, thereby identifying a ‘ceiling’ of neuroprotection unique to each therapeutic. A key concept is that the severity of the OGD insult must be matched by commensurate increases in the ‘strength’ of the neuroprotective approach. Finally, the ‘last man standing’ concept means that achieving efficacy meant comparing within-, between- and outside-classes of drugs, first alone and then in combinations, and evaluating dose-responses to arrive at the highest degree of efficacy possible.

For the adverse effect component of the therapeutic index, multi-electrode arrays (MEAs) can provide detailed non-invasive evaluation of drugs on neuron cultures or brain slices (acute, organotypic or any other brain dispersion such as 3D neurospheres or organoids). Neuronal function can be interrogated non-invasively over the lifetime of the cultures, providing a detailed profile of synaptic neuron and network activity (over 100 different descriptors of activity can be examined). Rather than producing a live/dead terminal assay at a single time-point, MEAs allow extended investigations into whether neuroprotection truly persists. MEAs represent a powerful complement to more conventional toxicity assays, Ca^2+^-spiking assays and patch-clamp electrophysiology, which can be insensitive/invasive/low-throughput. Plate-reader based MEA formats allow higher-throughput capability. MEAs allow the evaluation of the functional impact of ischemic-like insults [230,231,232] or drugs [233] on neurons and network activity. We combined both types of evaluations, in which live/dead assays were complemented by using MEAs to establish if an ostensible neuroprotective preconditioning treatment truly resulted in a return to normal synaptic activity by monitoring electrical activity non-invasively over 10–12 days before, during and after application of a neurotherapeutic modality ± insults [101]. Summarizing, the evaluation of neuronal adverse effects can be evaluated primarily using MEAs, although we foresee MEAs also providing a functional assessment of efficacy, thereby providing therapeutic indices through MEAs alone. This represents a powerful approach to help understand how a therapeutic might alter and preserve neuronal activity in vivo.

## 6. How Do Past and Current Prospects Fit Within the Proposed Neuroprotective Framework?

We now consider the proposed neuroprotective framework in a reverse-translational manner for therapeutics currently undergoing evaluation in clinical trials, as well as in animal models. It was pointed out 15 years ago that most drugs that failed in clinical trials were abandoned by sponsors, rather than back-tested in laboratory models, so that explanations for failures were lacking, as was an improved path forward [53]. While numerous reviews indicated that these failures could be attributed to any one of a number of methodological issues in both clinical and preclinical work, the comprehensive body of work performed in vitro strongly suggests that these trials also would have failed due to a lack of universally applicable optimal selection of the neuroprotective modality.

Combinations with at least one drug targeting excitotoxicity improved neuroprotection in vivo, but only with careful selection of the individual components. In one study, a combination of magnesium sulfate, melatonin and minocycline was chosen based on so-called drugability criteria such as therapeutic target, cost, availability, efficacy, administration and safety criteria, and tested in models favored by STAIR. No neuroprotection was observed, even with repeated decreases in model stringency [234]. If neuron culture data are representative, this failure likely reflects weak efficacy by memantine due to its lower potency, and less relevant biological signaling being targeted by the other two drugs. Other studies combining an NMDAR antagonist with different drugs improve efficacy [235,236,237,238,239,240], providing a template of what might be possible if a more rigorous framework was followed.

Another instructive example of several of the concepts advanced here is the high-profile phase III failure of the antioxidant NXY-059 in 2007 that set off a ‘nuclear winter’ [241] in translational stroke research, even though serious quality-based limitations in preclinical animal model studies were retrospectively identified [24,242,243]. Due to its poor BBB permeability, and poor cellular penetration [244,245], this drug has been classified as vasculoprotective [246], and cannot be considered as a neuroprotective drug. Back-testing of NXY-059 after clinical trials revealed no neuroprotection against OGD in human stem cell derived neurospheres [247]. An earlier study indicated modest neuroprotection against OGD in brain slice explants [248]. NXY-059 is a derivative of α-phenyl-N-tert-butyl nitrone (PBN), which has far better cell penetrability and is more lipophilic [244]; a stilbazulenzl nitrone (STAZN) is a much more potent antioxidant than either agent, reduces infarct volumes and is far more potent than NXY-059 (reviewed in [249]). Individual/comparative evaluations of efficacy against OGD or similar insults in vitro of the type advocated for in this review would have been useful in prioritization (and de-risking) within this class of antioxidants. As another example, high-dose human albumin can be regarded a pleiotropic drug which nonetheless failed in clinical trial [250]; again, although labeled a neuroprotectant, in vitro data demonstrating neuroprotection is absent, as far as we are aware.

What about current prospects? A very recent review provides an overview of a wide range of therapeutics currently in clinical trials, citing some failures, but also some successes, for which larger clinical trials are suggested [15]. However, in almost every case cited in that review [15], caution is merited, when considered against the neuroprotective framework, primarily due to concerns about efficacy. One example cited [15] as promising is uric acid, which met its primary outcome (in 67% of patients versus 48% in placebo) in the URICO-ICTUS trial; however, in vitro neuron culture data shows that neuroprotection by uric acid declines with increasing dose of exogenous glutamate (even though the glutamate insult was not supra-lethal) [251], thereby not meeting our criteria required for sufficient efficacy. Moreover, uric acid does not cross the blood–brain barrier [252], and cannot be considered as a neuroprotective candidate in the conventional definition (several other drugs within that review [15] also do not fit the neuroprotective definition). We have outlined concerns above with poor efficacy associated with verapamil and nerinetide. Neu2000 showed no efficacy in a phase II trial (SONIC), although a phase III trial (RODIN) is in progress. Neu2000—a derivative of aspirin and sulfasalazine—provides better neuroprotection than either drug alone against exogenous NMDA in cortical neuron culture, but only at relatively high concentrations (IC_50_ = 35 µM) [253]. Remote ischemic conditioning has failed to demonstrate benefit in clinical trials; post-conditioning is more challenging to study in vitro but, if efficacy expected is generally analogous to preconditioning, the body of work performed by ours and other laboratories in vitro suggest insufficient efficacy. It is doubtful that any of those drugs would be effective subjected to increasingly severe OGD in neuronal cultures. Taken together, most recent and current drugs under potential consideration generally lack even the most rudimentary proof of actual neuroprotection in vitro. It may be argued that demonstrations of efficacy in animal models of ischemia pre-empt the necessity of demonstrating any neuroprotection in vitro, let alone the strong efficacy being argued for in this review. However, given the extensive body of work to date, drugs or modalities without neuroprotective targeting have not translated to the clinic. Moreover, with the potential ability to deliver a drug to the infarct, various ancillary properties that drugs may possess may be of far less value than possessing direct neuroprotective ability. Moreover, this is not enough: the in vitro data strongly suggest that a much more aggressive approach to neuroprotection is required than previously appreciated.

## 7. Conclusions

**Figure 2:**
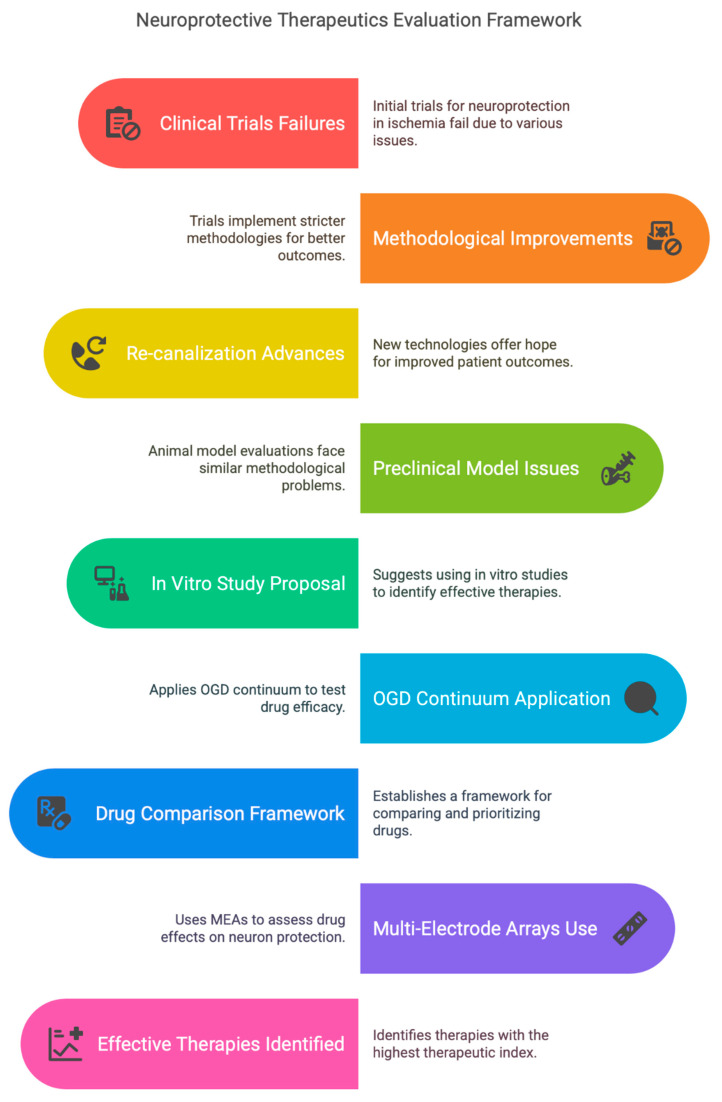
A schematic outlining the rationale used in devising the neuroprotective framework being proposed to prioritize candidates for translation to preclinical animal models. For the past few decades, numerous reviews providing critical insight into preclinical/clinical methodology or neuroprotection in cerebral ischemia frequently concluded by citing new and emerging neuro- or cyto-protective therapies, often in an optimistic (or at least hopeful) context. Due to the absence of a gold standard at the clinical level, as well as the preponderance of preclinical animal model ‘successes’ of therapeutics targeting numerous cellular signaling pathways, considerable confusion exists about which biological targets should be prioritized. Even with a better resolution of what signaling pathway(s) to target, considerable uncertainty also exists about how to choose among the many possibilities that now exist the best candidates for translation. What has been missing is a strategy to de-risk the translational pipeline. Adopting a ‘success from failure’ mindset allows the limits of efficacy and adverse effects of each therapeutic to be defined earlier in the translational process, thereby providing experimentally verified conditions to achieve the highest therapeutic index possible.

Given the considerable uncertainty that still exists in the neuroprotection field, the focus of this review was necessarily on maximizing efficacy, in an effort to understand what the biological target is under increasingly severe OGD conditions, with little regard for drugability at this stage. As reviewed here, neuroprotection under these conditions requires an increasingly aggressive approach, much more so than has conventionally been realized. Anti-excitotoxic based antagonism was by far the most neuroprotective in vitro; however, this was not fully comprehensive and, in slices in particular, protection was not absolute under more stringent ischemia-like models. If strong anti-excitotoxic modalities continue to maintain high relevance in the face of future studies, intra-arterial delivery of drugs targeting excitotoxity should be more permissive in retaining the higher potency and dosing required compared to systemic delivery. Intra-arterial delivery may even allow sufficient efficacy for fringes of the ischemic core to be tackled, if applied early enough, which again requires a strong therapeutic. Nonetheless, the requirement for an increasingly stronger therapeutic(s), particularly as the harshness of the insult increases, will likely produce unacceptably low therapeutic indices due to high adverse effects. Thus, concessions in the nature/potency/dose of the therapeutic(s) will be necessary to minimize adverse effects to an acceptable level, which will entail a penalty as to how severe an insult can be withstood while preserving efficacy. The challenge has always been a trade-off between minimizing adverse effects while still trying to maintain as high a degree of efficacy as possible. For the future, the goal is to be able to tailor a neuroprotective response to provide a sufficient therapeutic index that matches the severity of the ischemic insult. Patients are literally losing their minds after experiencing cerebral ischemia. Scientists, clinicians and caregivers have been incredibly frustrated with the unremitting failures, metaphorically losing their minds. The neuroprotective framework offered in this review provides a strategy aimed at preventing this from happening (Figure 2) which, combined with methodological and technical advances in preclinical animal model testing and clinical trials, may usher in a new era in protecting the brain from stroke.
